# A robustness-first cross-disease framework supports a candidate shared microbial redox axis and disease-specific metabolic divergence in colorectal cancer, Crohn’s disease, and liver cirrhosis

**DOI:** 10.1128/msystems.00836-26

**Published:** 2026-09-14

**Authors:** Kartika Sahu, Palok Aich

**Affiliations:** 1 School of Biological Sciences, National Institute of Science Education and Research (NISER), Khurda, Odisha, India; 2 Homi Bhabha National Institute 232022, Mumbai, Maharashtra, India; Istanbul Medipol Universitesi Uluslararasi Tip Fakultesi, Istanbul, Türkiye; Marmara University, Istanbul, Türkiye

**Keywords:** gut microbiome, maximin optimization, multi-omics integration, cross-disease inference, dysbiosis architecture, machine learning, SHAP

## Abstract

Gut microbiome dysbiosis is associated with colorectal cancer (CRC), Crohn’s disease (CD), and liver cirrhosis (LC), yet whether these diseases share conserved microbial vulnerabilities remains unresolved. Analytical pipeline choices alone can shift apparent performance from near-zero to near-perfect on identical data, rendering cross-disease comparison unreliable. Here, in a secondary cross-sectional analysis of public data sets, maximin optimization is introduced as a proposed pipeline-selection criterion guaranteeing worst-case performance across all tasks simultaneously. Benchmarking 1,152 preprocessing-model configurations across six tasks using gut metagenomics and serum metabolomics from CRC, CD, and LC reveals a candidate 19-species microbiome signature in which *Firmicutes* bacterium CAG:41 is the sole threshold-stable cross-disease taxon, depleted in all three diseases. Microbiome pathway enrichment converges on sulfur-selenium redox metabolism and B-vitamin biosynthesis, while host metabolomic responses are predominantly disease-specific. CD and LC are dominated by single discriminative taxa; CRC requires community-level integration. Exploratory external evaluation was adequately powered only for CRC (AUC = 0.769, 95% bootstrap CI 0.678–0.849); CD and LC assessments were exploratory only.

**IMPORTANCE:**

Cross-disease microbiome comparison has lacked a principled analytical foundation: arbitrary preprocessing choices can shift apparent classification performance from near-random to near-perfect on identical data, making biological conclusions unreliable when pooled across diseases. Maximin optimization addresses this by providing a decision-theoretic guarantee that every classification task contributes valid signal, enabling the first analytically controlled cross-disease comparison of gut metagenomics and serum metabolomics across three major gut-associated diseases. The identification of *Firmicutes* bacterium CAG:41 as the sole threshold-stable cross-disease taxon, harboring predicted functions in sulfur-selenium metabolism, offers a concrete target for experimental characterization and prospective screening validation. The two-layer dysbiosis architecture—universal microbial vulnerability with disease-specific host metabolic responses—provides a conceptual template for cross-disease microbiome study design in other gut-associated conditions, pending prospective confirmation.

## INTRODUCTION

The human gut microbiome functions as a metabolically active organ, supplying essential cofactors, modulating immune homeostasis, maintaining epithelial barrier integrity, and regulating redox balance through its collective biochemical activity (1–4). Disruption of this ecosystem, referred to as dysbiosis, has been independently associated with colorectal cancer (CRC), Crohn’s disease (CD), and liver cirrhosis (LC) (5–7). CRC is the third most common cancer globally, with 1,926,425 incident cases and 904,019 deaths recorded in 2022 (8). CD, a major subtype of inflammatory bowel disease (IBD), contributes to a global IBD incidence of 8.77 cases per 100,000 population as of 2021 (9). Liver cirrhosis, the end-stage of multiple chronic liver diseases, affected an estimated 58.4 million people globally in 2021 (10). Despite affecting anatomically distinct organs, all three diseases interface with the gut microbiome through the gut-liver axis, and large-scale metagenomic surveys have revealed extensive taxonomic and functional alterations across each condition (5–7).

A central unresolved question is whether these diseases share conserved microbial vulnerabilities exploitable for cross-disease screening or therapeutic intervention, or whether their microbial associations are fundamentally disease-specific. Multiple studies have documented the sensitivity of microbiome ML performance to preprocessing and modeling choices. Decisions regarding scaling, normalization, feature selection, and model architecture can substantially alter apparent predictability (11–14), and most studies report only peak performance from arbitrarily chosen pipelines (5, 15).

Microbial composition alone further provides an incomplete picture. Gut microorganisms exert influence on host physiology primarily through metabolic outputs, and identical compositional dysbiosis may produce divergent host metabolic consequences depending on organ-specific physiology and disease context.

Three hypotheses are tested here. First, that robustness-first optimization will expose conserved biological signals that arbitrary pipeline selection obscures or distorts. Second, that the architecture of disease-associated dysbiosis may not be uniform; some diseases may be dominated by few highly discriminative taxa while others require community-level integration with direct potential for diagnostic platform design. Third, even if conserved microbial perturbations exist across diseases, their downstream host metabolic consequences may be disease-specific rather than universal, reflecting the filtering effect of organ-specific physiology.

To test these hypotheses, a robustness-first analytical framework centered on maximin optimization is developed—a proposed criterion from decision theory (16)—that selects the pipeline maximizing worst-case performance across all tasks simultaneously, guaranteeing that every disease and omics layer contributes valid signal to cross-disease comparison. A total of 1,152 preprocessing-model configurations are benchmarked across six binary classification tasks spanning CRC, CD, and LC using gut metagenomics and serum metabolomics under rigorously nested cross-validation. SHapley Additive exPlanations (SHAP)-based feature attribution (17) and pathway over-representation analysis are applied across both omics layers. To the best of our knowledge, this is the first application of maximin optimization as a pipeline-selection criterion in multi-omics-based multi-disease informatics.

## RESULTS

### Analytical choices dominate apparent predictability

**Fig 1:**
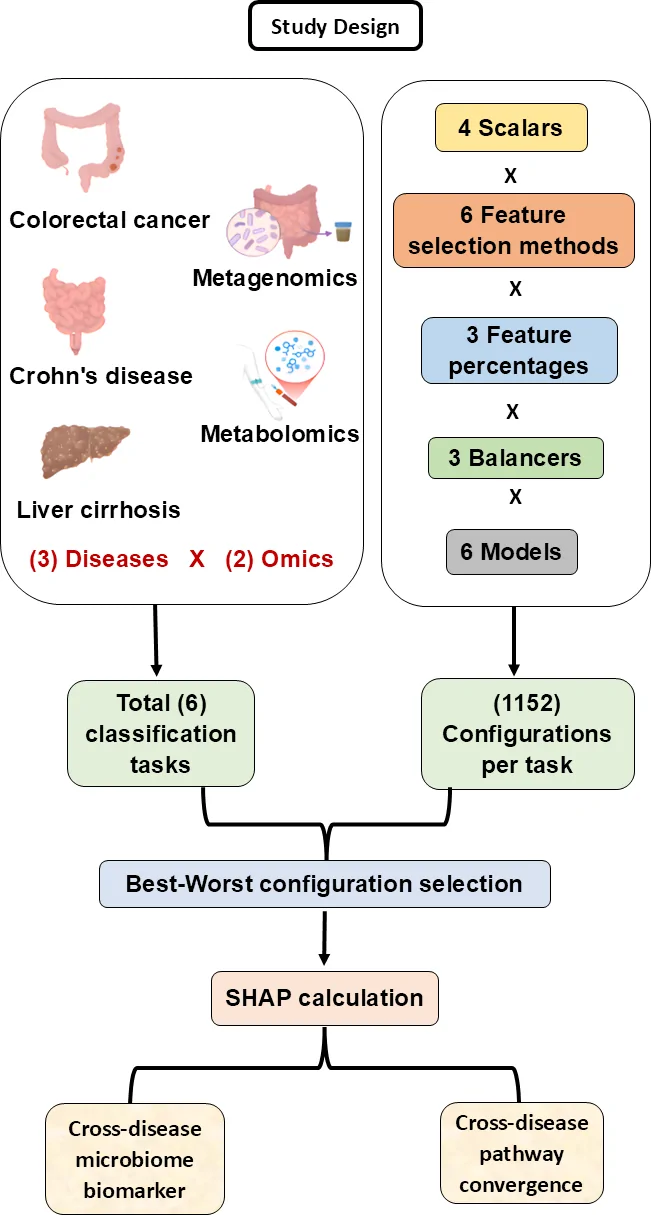
Overview of the robustness-first, cross-disease analytical framework. Microbiome and serum metabolomics data from CRC, CD, and LC cohorts were analyzed across six binary classification tasks (three diseases × two omics platforms) using systematic benchmarking of 1,152 preprocessing-model configurations with rigorously nested cross-validation. A maximin robustness criterion identified a unified pipeline optimized for worst-case performance across all diseases. SHAP-based feature attribution and pathway over-representation analysis revealed conserved microbial vulnerabilities and cross-disease biomarkers.

**Fig 2:**
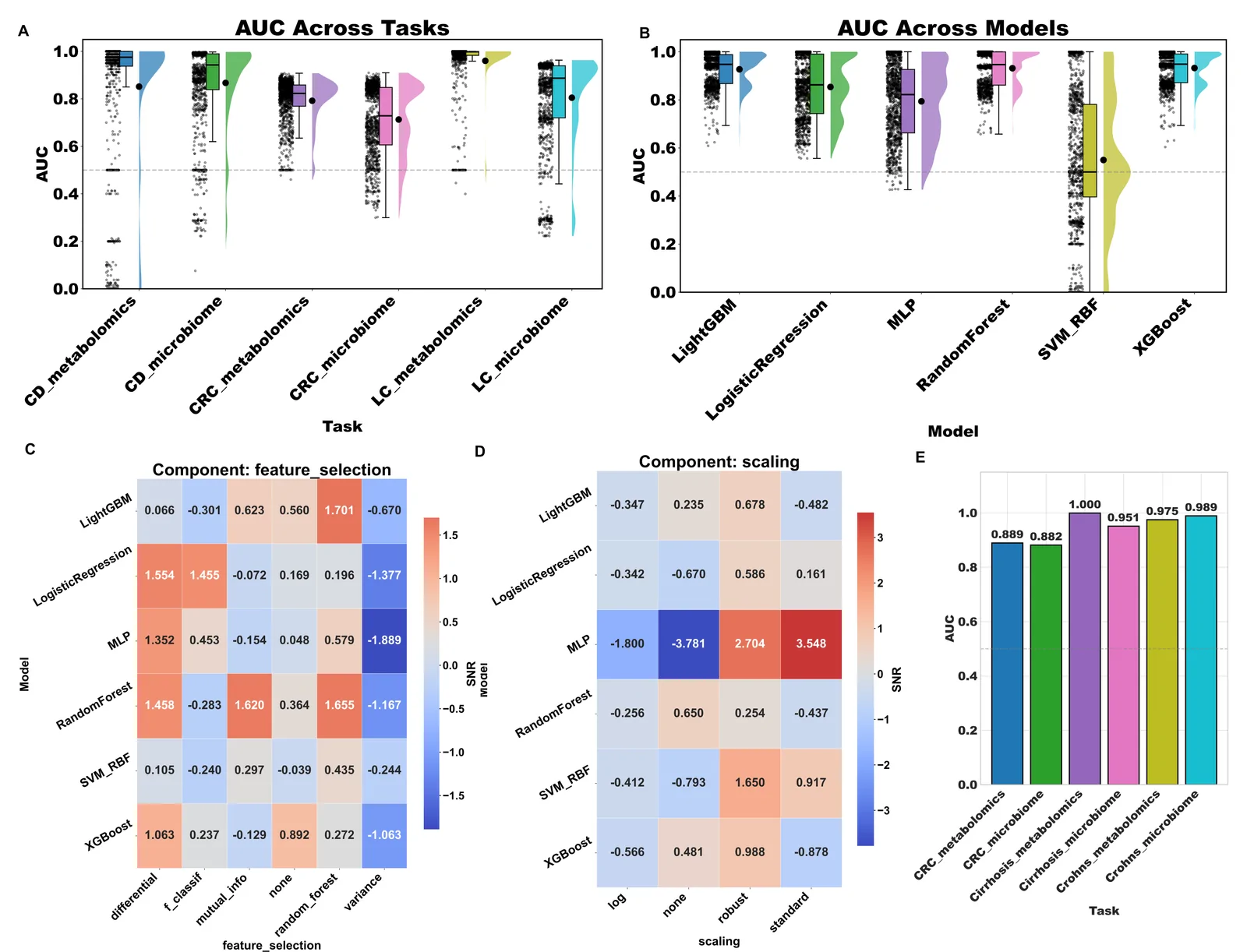
Systematic benchmarking reveals analytical instability dominates apparent microbiome predictability. (**A**) Raincloud plots showing AUC distribution across six classification tasks (CRC, CD, LC × microbiome, metabolomics). Each plot displays the probability density distribution (cloud), individual data points (rain), interquartile range (IQR bar), median (horizontal black line within bar), and mean (black dot). Within-task AUC ranges were substantial: CRC microbiome (0.30–0.91), CD microbiome (0.08–0.997), LC microbiome (0.22–0.96), CRC metabolomics (0.46–0.91), CD metabolomics (0.00–1.00), and LC metabolomics (0.40–1.00), demonstrating that analytical choices profoundly shape apparent disease predictability. (**B**) Raincloud plots showing AUC distribution across six machine learning models: random forest (RF), XGBoost, LightGBM, logistic regression (LR), support vector machine with radial basis function kernel (SVM-RBF), and multilayer perceptron (MLP). Plot structure identical to panel A. Tree-based ensemble models (XGBoost, LightGBM, RF) achieved higher median performance than kernel-based and neural network approaches across most tasks. (**C**) Heatmap showing signal-to-noise ratio (SNR) analysis of model-feature selection method interactions. Rows represent machine learning models; columns represent feature selection methods (no selection, variance threshold, ANOVA F-test, mutual information, random forest importance, Mann-Whitney *U* test) evaluated at three retention levels (10%, 30%, 50%). Color intensity indicates SNR magnitude (red = positive SNR, consistent improvement; blue = negative SNR, consistent degradation). (**D**) Heatmap showing SNR analysis of model-scaling method interactions. Rows represent machine learning models; columns represent scaling strategies (no scaling, standard scaling, robust scaling, log scaling). Multilayer perceptrons exhibited pronounced sensitivity to feature scaling (maximum SNR difference = 7.33), while tree-based models showed relatively stable performance across scaling methods. (**E**) Bar plot showing performance of the robustness-optimized pipeline (XGBoost classifier, standard scaling, random forest feature selection, no class balancing) across all six tasks.

To quantify the impact of analytical decisions on apparent disease predictability, 1,152 distinct preprocessing-model configurations were evaluated across six classification tasks using nested cross-validation, spanning four scaling strategies, six feature selection methods, three class-balancing approaches, and six classifiers (Fig. 1). Across all tasks, predictive performance varied widely with analytical configuration (Fig. 2A). Within-task AUC ranges were CRC microbiome (0.30–0.91), CD microbiome (0.08–0.997), LC microbiome (0.22–0.96), CRC metabolomics (0.46–0.91), CD metabolomics (0.00–1.00), and LC metabolomics (0.40–1.00). The extreme range in CD metabolomics likely reflects instability due to small sample size (*n* = 40) and should be interpreted with particular caution.

Tree-based models achieved higher median performance than kernel-based and neural network approaches (Fig. 2B). Signal-to-noise ratio (SNR) analysis revealed strong preprocessing-model interactions: multilayer perceptron (MLP) exhibited pronounced sensitivity to feature scaling (maximum SNR difference = 7.33; Fig. 2D), while variance-based feature selection predominantly degraded performance across all models (SNR range: −0.24 to −1.89; Fig. 2C; Fig. S2).

### Robustness-first optimization identifies a unified pipeline

No single configuration achieved consistently high performance across all tasks (Fig. S3). To identify a single unified pipeline valid for cross-disease comparison, maximin optimization was applied, selecting the configuration maximizing minimum AUC across all six tasks simultaneously. The maximin-optimized pipeline achieved minimum cross-task AUC of 0.882 (permutation *P* < 0.01; Fig. 2E).

To assess the robustness of this selection, three alternative criteria were evaluated across all configurations present in all six tasks. Mean-AUC selection identified a different pipeline (XGBoost, log scaling, f_classif, synthetic minority over-sampling technique [SMOTE]) with minimum cross-task AUC of 0.872. Median-AUC selection identified a further distinct pipeline (LightGBM, log scaling, variance-based feature selection, SMOTE) with a minimum of 0.809. Pareto-optimal multi-objective selection, which simultaneously minimizes worst-case and mean regret, selected the identical pipeline as maximin (minimum AUC = 0.882) (Table S1).

External validation was performed using the maximin-selected pipeline applied to European cohorts without retraining. Three evidential tiers are distinguished. Tier 1 (primary evidence base): internally validated performance under fivefold nested cross-validation with inner threefold hyperparameter tuning; all biological conclusions in this study derive from this tier. Tier 2 (adequately powered external evaluation, CRC only): in an independent French cohort (PRJEB6070; *n* = 53 disease, 61 healthy), external AUC = 0.769 (95% bootstrap CI 0.678–0.849). Tier 3 (exploratory, not suitable for generalization inference): for CD (PRJEB2054; *n* = 4 disease, 14 healthy), AUC = 0.875 (95% bootstrap CI 0.66–1.000); the lower bound of 0.66 spans near-chance performance. For LC (PRJEB38481; *n* = 90 disease, no healthy controls), AUC is not computable; positive prediction rate = 0.722 (95% bootstrap CI 0.622–0.811). Tier 3 results are retained for transparency only.

For LC metabolomics (*n* = 874 disease, 29 healthy; approximately 30:1 imbalance), the maximin-selected pipeline (no class balancing) achieves mean-of-folds ROC-AUC = 0.999, PR-AUC = 1.0000, balanced accuracy = 0.9500. Repeated downsampling to 1:1 and 3:1 case:control ratios (*N* = 100 iterations each) showed robust performance: 1:1 median ROC-AUC = 0.9944, PR-AUC = 0.9952, balanced accuracy = 0.9333; 3:1 median ROC-AUC = 0.9961, PR-AUC = 0.9988, balanced accuracy = 0.9333. Balanced accuracy did not degrade under stronger balancing, ruling out majority-class bias. Maximin pipeline selection was unchanged when LC metabolomics was excluded from optimization (bottleneck task was CRC microbiome in both conditions).

Per-sample age, sex, and BMI were available for all three microbiome training cohorts. Covariate-complete sensitivity analyses were performed by running the maximin pipeline on the identical covariate-complete subset twice: (i) microbiome features only; (ii) microbiome features with age, sex, and BMI appended. ΔAUC was within ±0.022 across all diseases and all retention levels, with no consistent directional effect (Table S2).

### Distinct dysbiosis architectures

**Fig 3:**
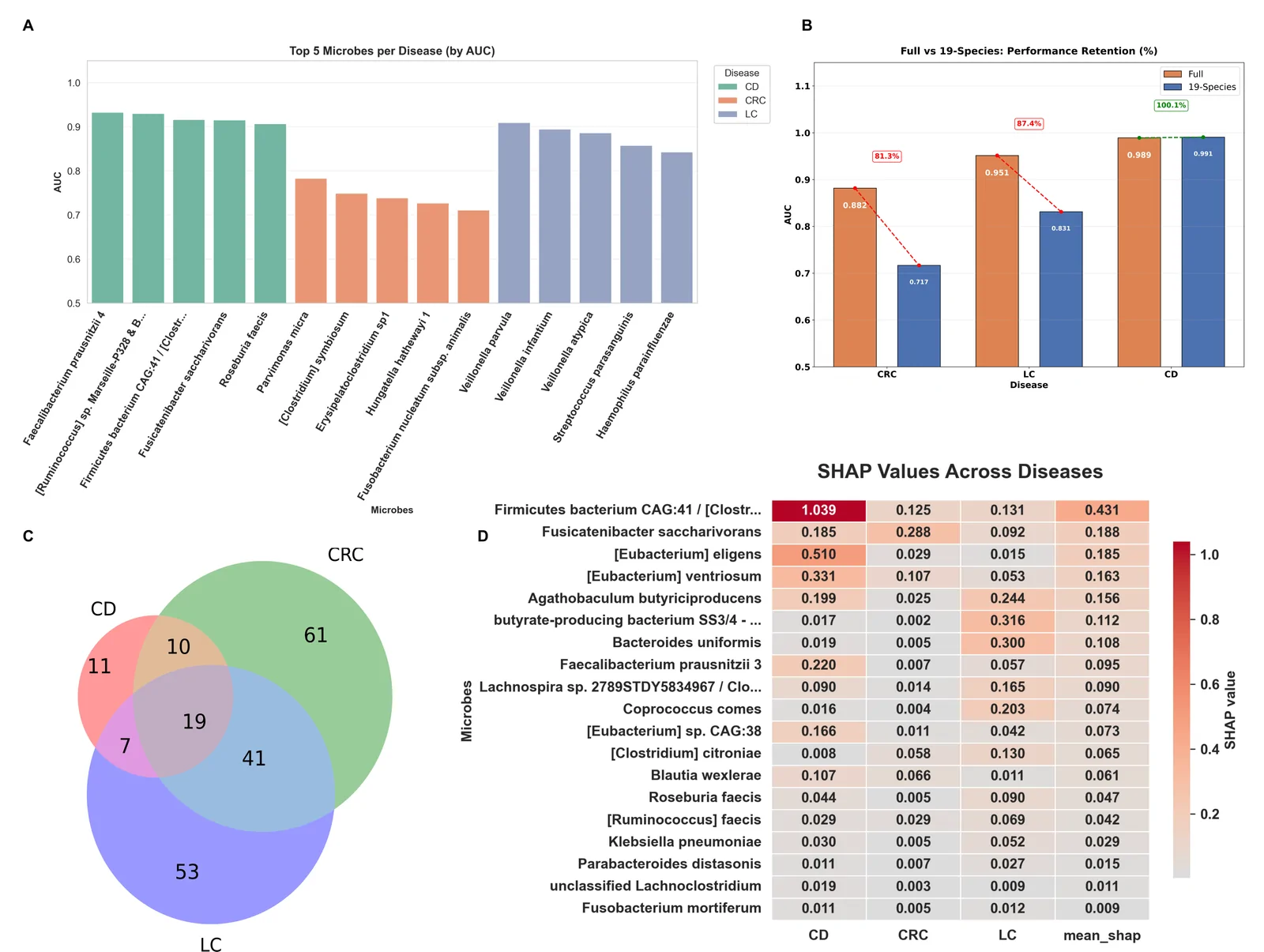
Dysbiosis architectures and conserved cross-disease microbiome signature. (**A**) Bar plot showing the top five discriminative microbial species from univariate analysis for each disease, ranked by single-feature AUC. CD and LC exhibited “dominant” dysbiosis architectures with single taxa approaching multivariate model performance (*Faecalibacterium prausnitzii* AUC = 0.933 ± 0.042 in CD; *Veillonella parvula* AUC = 0.91 ± 0.036 in LC). In contrast, CRC displayed “distributed” dysbiosis with no single species exceeding AUC = 0.80 (top taxon: *Parvimonas micra* AUC = 0.783 ± 0.021), requiring multi-species integration for optimal classification. (**B**) Bar plot comparing performance of full-feature models versus 19-species signature models using the robustness-optimized pipeline across three diseases. For each disease, paired bars show AUC for the full 1,990-species model (orange bars) and the 19-species conserved signature model (blue bars), with performance difference indicated. Signature species retained high performance in CD and LC (dominant architectures), with larger reduction observed in CRC (distributed architecture), mirroring univariate signal decomposition patterns. (**C**) Venn diagram showing intersection of SHAP-identified predictive species across CRC, CD, and LC. The conserved cross-disease signature comprises 19 species with non-zero SHAP importance in all three diseases (central intersection), alongside disease-specific features. Numbers indicate species counts in each region. (**D**) Heatmap showing SHAP values of the 19 conserved signature species across three diseases. Rows represent individual species (ranked by mean SHAP value); columns represent diseases (CRC, CD, LC). Color intensity indicates SHAP magnitude (contribution to disease prediction). *Firmicutes bacterium CAG:41* exhibited the highest mean SHAP value (0.431) and was consistently depleted across all diseases (relative abundance reductions: CD −87.5%, CRC −58.0%, LC −13.1%). Disease-specific high-ranking contributors included *P. micra* (CRC), *F. prausnitzii* (CD), and *Veillonella infantium* (LC).

To characterize how predictive signal is distributed across individual taxa within each disease, single-feature AUCs were computed for all 1,990 detected microbial species using logistic regression under fivefold stratified cross-validation. Univariate analysis revealed two distinct signal architectures (Fig. 3A). In CD and LC, individual taxa dominated prediction: *Faecalibacterium prausnitzii* achieved AUC of 0.933 ± 0.042 in CD; *Veillonella parvula* reached 0.91 ± 0.036 in LC with several additional *Veillonella* species exceeding 0.85. In CRC, signal was distributed: the strongest predictor, *Parvimonas micra* (AUC = 0.783 ± 0.021), performed substantially below the multivariate model (0.882), with no species exceeding 0.80.

CD and LC showed strongly right-skewed AUC distributions (skewness = 1.58 and 1.80), whereas CRC exhibited weaker skewness (1.33), consistent with signal distributed across taxa rather than concentrated in dominant species (Fig. S4). Based on these criteria, where no single taxon exceeds an AUC of 0.80 for CRC with low skewness and single taxa approach multivariate model performance for CD and LC with higher skewness, CRC is classified as a distributed dysbiosis architecture, while CD and LC are classified as dominant dysbiosis architectures.

### Candidate 19-species cross-disease signature

To identify species contributing consistently across all three diseases, species with non-zero SHAP importance in all three maximin-optimized disease models were extracted. Nineteen species had non-zero SHAP importance across all three diseases, comprising the candidate cross-disease panel (Fig. 3C and D). To assess stability, we applied increasingly stringent SHAP thresholds to the cross-disease intersection. At top 25% and mean SHAP thresholds, *Firmicutes* bacterium CAG:41 (also designated [*Clostridium*] sp*.* 2789STDY5834935 and sp*.* 2789STDY5608853; mean SHAP = 0.431) was the sole species retained across all three diseases, and it was depleted across all diseases relative to their controls (−87.5% in CD, −58.0% in CRC, −13.1% in LC, Mann-Whitney U, FDR-corrected *P* < 0.05). The remaining 18 species satisfied only the inclusive non-zero criterion and are therefore described as a hypothesis-generating candidate panel requiring prospective validation. Functional annotations (computationally predicted, requiring experimental validation) indicate genes for selenocompound metabolism, cysteine biosynthesis, and B-vitamin pathways (18).

Disease-specific contributors included *P. micra* (CRC), *F. prausnitzii* (CD), and *V. infantium* (LC). Models using only the 19-species signature retained high performance in CD and LC, with a larger reduction in CRC (Fig. 3B), mirroring dominant versus distributed architectures.

### Disease-specific metabolomic signatures

SHAP-based feature attribution was applied to serum metabolomics data using the maximin-optimized pipeline to characterize disease-specific metabolic signatures. In CRC, top-ranked metabolites included histidine, glyceraldehyde, malonic acid, and maleic acid. LC was characterized by taurine, dehydroisoandrosterone sulfate, glycerol-3-phosphate, and hydroxyphenyllactic acid. In CD, the most influential metabolites were sucrose, dimethylarginine, and 3-hydroxybutyrylcarnitine. No metabolite ranked consistently across diseases.

### Conserved sulfur-selenium enrichment in microbiome pathways

**Fig 4:**
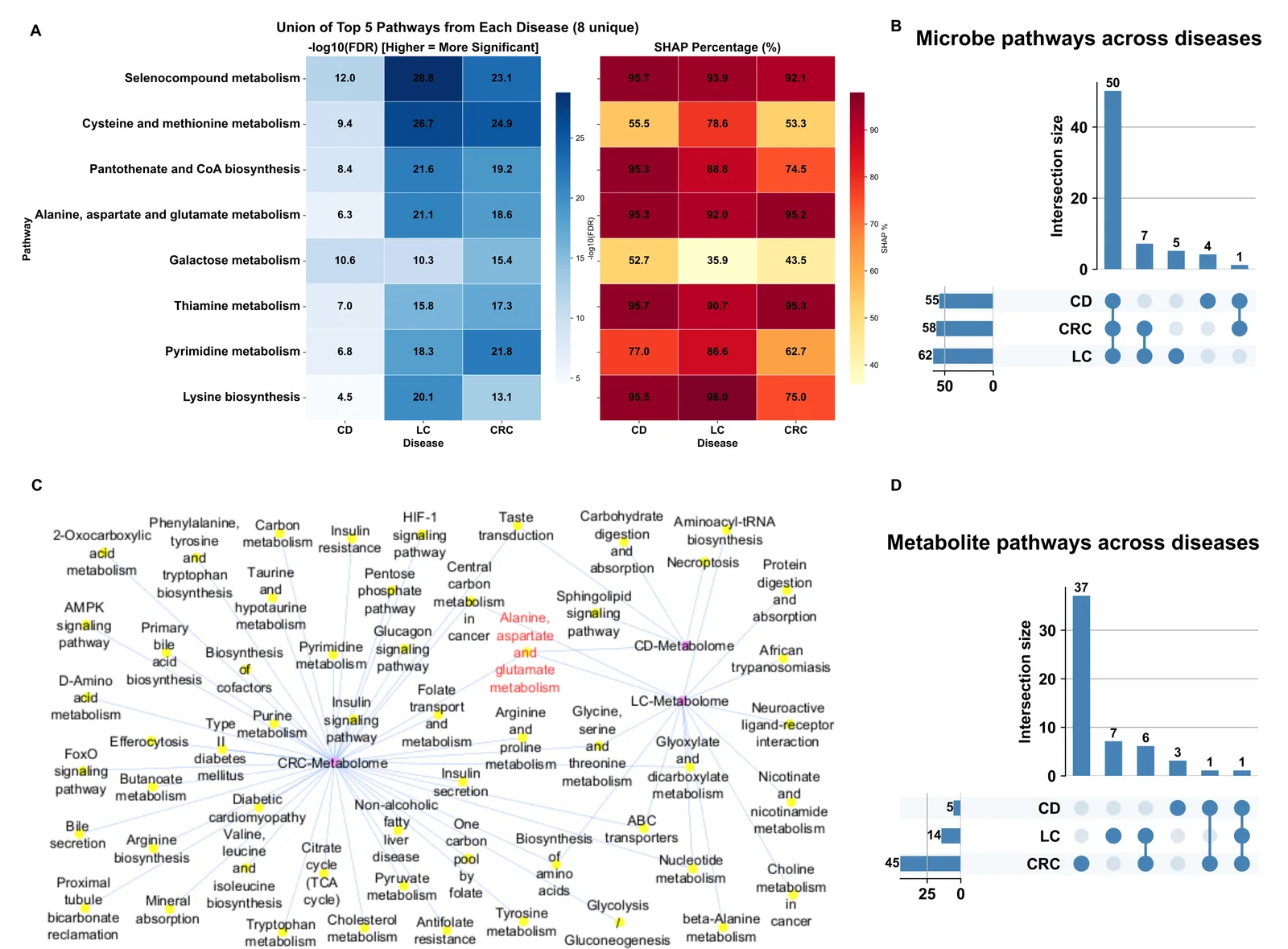
Functional convergence and omics asymmetry. (**A**) Heatmap showing enrichment analysis of microbiome pathways across diseases. Rows represent the union of the top five enriched pathways from each disease (eight unique pathways total after removing duplicates). Columns display −log₁₀(FDR-adjusted *P* value) for pathway enrichment (left section) and SHAP percentage contribution (right section) for each disease. Color intensity indicates magnitude. Cysteine and methionine metabolism ranked first in CRC and remained significantly enriched in CD and LC. Selenocompound metabolism ranked first in CD and LC and second in CRC, revealing conserved sulfur-selenium redox vulnerability across all three diseases. (**B**) UpSet plot showing convergence of enriched microbiome pathways across diseases. Horizontal bars (left) indicate the total number of enriched pathways per disease. Vertical bars show pathway intersection sizes; dots and connecting lines indicate which diseases share each intersection. The largest intersection represents 50 pathways shared across all three diseases (CRC ∩ CD ∩ LC), spanning amino acid metabolism, B-vitamin biosynthesis, nucleotide metabolism, DNA repair, and central carbon metabolism. (**C**) Network diagram showing metabolomic pathway enrichment across three diseases. Nodes represent enriched KEGG pathways and diseases. Edges connect pathways appearing in diseases. The network highlights extensive disease-specific metabolic reprogramming: CRC showed 45 enriched pathways (including central carbon metabolism in cancer, HIF-1 signaling, glycolysis/gluconeogenesis, nucleotide metabolism, and folate-mediated one-carbon metabolism); LC showed 14 pathways (protein digestion and absorption, aminoacyl-tRNA biosynthesis, choline metabolism, neuroactive ligand-receptor interaction); CD showed five pathways (carbohydrate digestion and absorption, necroptosis, sphingolipid signaling, taste transduction). Alanine, aspartate, and glutamate metabolism (highlighted in red color) represents the single pathway enriched across all three diseases in metabolomics. (**D**) UpSet plot showing convergence of enriched metabolomic pathways across diseases. Plot structure identical to panel B. In stark contrast to the 50 shared microbiome pathways, only one metabolomic pathway (alanine, aspartate, and glutamate metabolism) was common to all three diseases, revealing pronounced omics asymmetry wherein conserved microbial functional perturbations are filtered through disease- and organ-specific host physiology.

Pathway analysis of SHAP-weighted taxa identified consistent enrichment of sulfur-selenium pathways across diseases (Fig. 4A). Cysteine and methionine metabolism (sulfur-containing amino acids) ranked first in CRC (FDR < 0.001) and remained significant in CD and LC (FDR < 0.05). Selenocompound metabolism ranked first in CD and LC. Of all Kyoto Encyclopedia of Genes and Genomes (KEGG) pathways tested, 50 were shared across diseases (Fig. 4B), spanning amino acid metabolism, B-vitamin biosynthesis, and nucleotide metabolism.

### Metabolomic pathway responses are predominantly disease-specific

In contrast, metabolomic pathways were predominantly disease-specific. CRC showed 45 enriched pathways (central carbon metabolism, hypoxia-inducible factor-1 signaling, glycolysis); LC showed 14 (protein digestion, choline metabolism, aminoacyl-tRNA biosynthesis); CD showed 5 (alanine/aspartate/glutamate metabolism, necroptosis, sphingolipid signaling) (Fig. 4C). Only alanine, aspartate, and glutamate metabolism was the single pathway shared across all diseases in metabolomics and also in both omics layers (Fig. 4C and D; Fig. S5). Because SHAP-derived features were used as the foreground set for over-representation analysis in both metagenomics and metabolomics data, these pathway findings are pipeline-dependent and should be interpreted accordingly.

Platform heterogeneity and limited KEGG annotation coverage across metabolomics data sets, with mapping rates of 71.4% for CRC (40 of 56 non-zero SHAP metabolites), 67.7% for LC (21 of 31), and 46.7% for CD (7 of 15), may partially explain this asymmetry between 50 shared microbiome pathways and one shared metabolomic pathway.

## DISCUSSION

Benchmarking 1,152 analytical configurations across six tasks spanning CRC, CD, and LC reveals how deeply pipeline choice confounds cross-disease inference, and demonstrates that maximin optimization resolves this by guaranteeing every task contributes valid signal.

### Maximin optimization as a proposed generalizable informatics method

The benchmarking revealed that no single conventional configuration achieved consistently high performance across all six tasks (Fig. S3). The maximin-optimized pipeline achieved a guaranteed minimum cross-task AUC of 0.882 (permutation *P* < 0.01), exceeding the minimum achieved by mean-AUC selection (0.872) and median-AUC selection (0.809), confirming that robustness-first criteria outperform central-tendency optimization on worst-case task performance. Notably, a Pareto-optimal criterion (minimizing worst-case regret relative to the task-specific best) selected the identical pipeline configuration, providing convergent support that the maximin-selected pipeline is not an artifact of the specific criterion but represents a genuinely robust configuration. Maximin is nonetheless proposed as one principled option within a broader family of valid robustness-first approaches rather than the uniquely correct criterion.

A specific preprocessing-model interaction with direct methodological implications for the informatics community is the pronounced sensitivity of multilayer perceptrons to feature scaling. This finding implies that deep learning approaches applied to microbiome data require more careful preprocessing validation than tree-based methods, which showed relative stability across preprocessing strategies. Variance-based feature selection predominantly degraded performance across models, with only a small number of isolated model-task combinations showing neutral effects, suggesting that low-variance taxa encode disease-relevant information that standard dimensionality reduction discards, a counterintuitive finding with implications for feature engineering in these types of data.

### Dominant versus distributed dysbiosis architectures

Consistent with our second hypothesis, univariate signal decomposition revealed two structurally distinct architectures. In CD and LC, predictive signal was concentrated in individual taxa: *F. prausnitzii* achieved a single-feature AUC of 0.933 in CD, approaching multivariate model performance, while *V. parvula* reached 0.91 in LC. These findings align with established associations linking *F. prausnitzii* depletion to mucosal barrier dysfunction (19) and *Veillonella* enrichment to oral-gut translocation in cirrhosis (7).

In contrast, CRC exhibited a distributed signal where no single species exceeded an AUC of 0.80, necessitating community-level integration. This distributed pattern may reflect systemic tumor-driven metabolic reprogramming that indirectly reshapes microbial niches through altered host physiology rather than through direct pathogen-epithelial interactions.

This architectural distinction carries potential implications for clinical diagnostic design that go beyond biological characterization. In diseases with dominant dysbiosis architecture, where a single taxon approaches multivariate model performance, targeted, low-cost diagnostic assays based on quantitative PCR or culture enrichment of a single marker organism are potentially viable. Such assays are potentially viable in resource-limited settings, pending prospective validation.

In contrast, the distributed dysbiosis architecture of CRC, where no single species exceeded an AUC of 0.80 and the community-level integration provided the critical margin, necessitates high-throughput metagenomics or panel-based sequencing for adequate diagnostic performance. This distinction may inform future clinical trial design for microbiome-based diagnostics. To our knowledge, this formal quantitative characterization of dominant versus distributed signal architectures is novel in the microbiome informatics literature and provides a replicable analytical template for classifying dysbiosis architecture in other diseases.

### Conserved sulfur-selenium redox vulnerability

Across all three diseases, microbiome pathway enrichment converged on sulfur and selenium metabolism (cysteine/methionine metabolism and selenocompound metabolism, both FDR < 0.05), an axis central to cellular antioxidant defense. These pathways supply sulfur for glutathione synthesis and support glutathione peroxidases (20, 21). This convergence is consistent with an earlier cross-disease meta-analysis by Duvallet et al. (22), who observed shared disease-associated taxa across multiple gut conditions and proposed that disruption of intestinal redox state may underlie cross-disease microbial responses rather than disease-specific pathogenic mechanisms, a hypothesis our pathway-level analysis under controlled analytical conditions is consistent with, providing mechanistic specificity to that earlier observation. Because pathway enrichment used SHAP-selected features as foreground, however, these findings are pipeline-dependent and should be considered as such. This convergence suggests that impaired microbiome-supported antioxidant capacity may represent an underappreciated dimension of dysbiosis, complementary to the short-chain fatty acid paradigm (23). Elevated oxidative stress is a shared feature of CRC, CD, and LC, contributing to DNA damage, epithelial barrier breakdown, and fibrogenesis (24–26), though whether microbial disruption of this axis is a cause or consequence of disease cannot be determined from cross-sectional data.

Beyond sulfur-selenium metabolism, we observed consistent enrichment of microbial pathways related to B-vitamin biosynthesis, including folate, riboflavin, thiamine, biotin, pantothenate, and vitamin B6. These cofactors are indispensable for redox enzymes, nucleotide synthesis, and amino acid metabolism, and the gut microbiome supplies a substantial fraction of host requirements (27, 28).

### Microbial dark matter dominates conserved signal

The strongest cross-disease signal resided in *Firmicutes* bacterium CAG:41, consistently the most influential species across all three diseases and markedly depleted in each. This reinforces growing recognition that clinically relevant microbiome signals often originate from poorly studied lineages (29). Database annotations in the Human Gut Microbiome Atlas indicate that CAG:41 harbors genes with predicted functions in selenocompound metabolism and cysteine biosynthesis, suggesting the genetic potential for these functions; whether these predicted capacities are active requires experimental validation through targeted cultivation and functional characterization.

### Omics asymmetry supports layered biological organization

Our third hypothesis predicted that microbial convergence would coexist with metabolomic divergence. Indeed, 50 microbiome pathways were shared across diseases compared with only a single metabolomic pathway, suggesting that conserved microbial functional perturbations may be filtered through organ-specific host physiology rather than translating into uniform systemic responses.

In CRC, metabolomic enrichment reflected aerobic glycolysis, HIF-1 signaling, and one-carbon metabolism, consistent with Warburg metabolism and biosynthetic demands of proliferating tumor cells (30–32). LC exhibited enrichment in choline metabolism and aminoacyl-tRNA biosynthesis, reflecting impaired bile handling and altered nitrogen metabolism (33–36). CD showed the most limited metabolomic footprint, with enrichment in amino acid metabolism, necroptosis, and sphingolipid signaling, supporting a model of localized, barrier-centered metabolic disturbance (37–40).

The only pathway enriched across both microbiome and metabolomics layers in all three diseases was alanine, aspartate, and glutamate metabolism. This hub directly links to the sulfur-selenium axis via glutamate as a precursor for glutathione synthesis (20); supports epithelial regeneration, hepatocyte turnover, and tumor growth via aspartate-fueled nucleotide synthesis (41); and integrates gut-liver metabolic communication through alanine-mediated nitrogen transfer and gluconeogenesis (42). Disruption of this hub could therefore simultaneously compromise redox defense, tissue repair, and energy metabolism, processes central to all three diseases.

However, identification of a single shared pathway could reflect chance overlap given multiple comparisons, and this finding requires independent confirmation. We also interpret the omics asymmetry with caution, as platform heterogeneity across metabolomics data sets may partially explain limited metabolomic convergence.

### A candidate two-layer architecture

**Fig 5:**
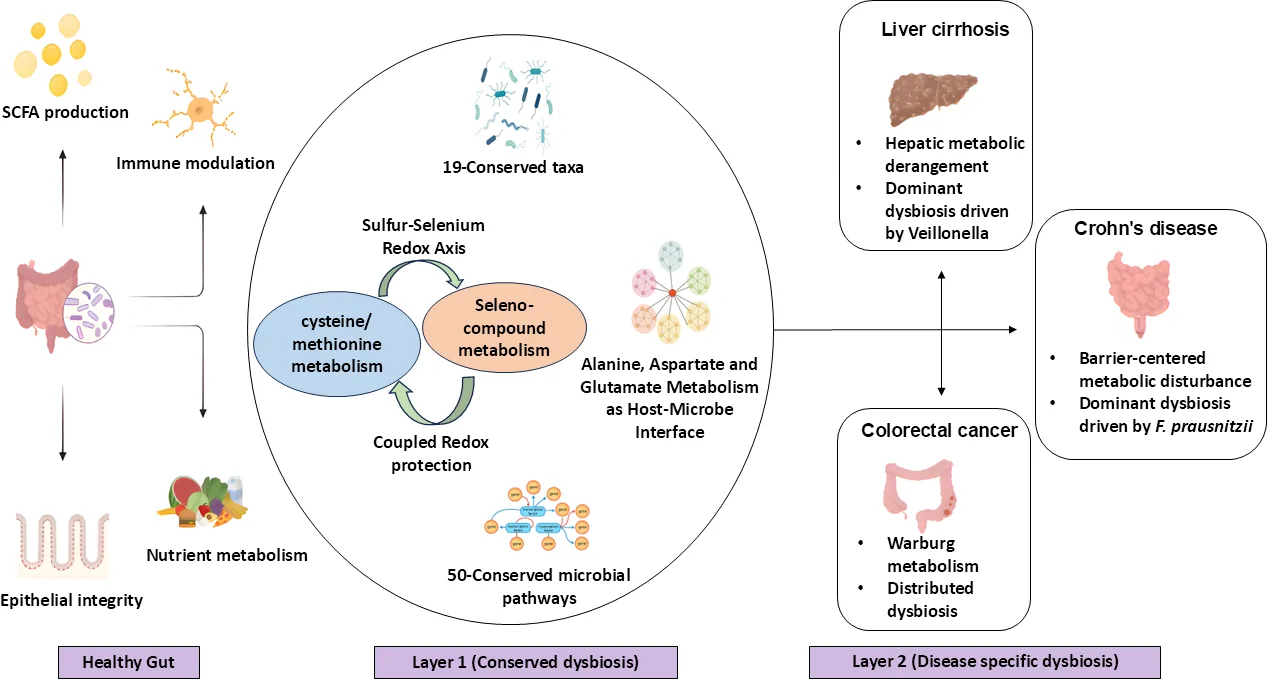
Two-layer architecture of gut dysbiosis. Integrated conceptual model unifying the two-layer dysbiosis architecture discovered through robustness-first cross-disease analysis. Healthy state: the gut microbiome performs various essential functions. Layer 1 (universal microbial vulnerability): conserved dysbiosis shared across CRC, CD, and LC, characterized by (i) disturbance of the 19-species candidate signature dominated by CAG:41; (ii) disruption of 50 shared microbiome pathways, particularly sulfur-selenium redox metabolism (cysteine/methionine metabolism, selenocompound metabolism) and B-vitamin biosynthesis; and (iii) perturbation of the universal host-microbe interface (alanine, aspartate, and glutamate metabolism). This layer represents fundamental ecosystem instability affecting redox defense, cofactor production, and metabolic communication. Layer 2 (disease-specific amplification): disease-specific pathological phenotypes arising from interaction between universal microbial vulnerability (layer 1) and local organ-specific contexts. In CRC, tumor-driven systemic metabolic reprogramming produces distributed dysbiosis architecture and extensive host metabolic alterations (Warburg metabolism, HIF-1 signaling, one-carbon metabolism). In CD, focal intestinal inflammation produces dominant dysbiosis architecture centered on *F. prausnitzii* with localized metabolic disruption (necroptosis, sphingolipid signaling). In LC, hepatic dysfunction and portal hypertension produce dominant dysbiosis architecture with *Veillonella* and liver-specific metabolic failure (choline metabolism, aminoacyl-tRNA biosynthesis).

The following clinical translation framework is proposed as a set of testable hypotheses derived from the current findings, rather than as validated clinical recommendations. The potential two-layer dysbiosis architecture identified in this study, a universal microbial vulnerability layer coexisting with a disease-specific host metabolic response layer, provides a conceptual framework for generating testable hypotheses. It has two complementary levels (Fig. 5).

At the universal layer, the candidate 19-species signature and the consistent sulfur-selenium enrichment motivate prospective evaluation of a pan-disease gut health screening approach. *Firmicutes* bacterium CAG:41 as the sole threshold-stable cross-disease taxon is a priority candidate. Microbiome-targeted interventions aimed at restoring selenocompound metabolism and cysteine biosynthesis represent testable hypotheses for future clinical trials, not established therapeutic recommendations.

At the disease-specific layer, the distinct metabolomic signatures support differential diagnostic hypotheses. This two-stage architecture suggests a framework for future prospective validation study design.

We acknowledge that the candidate signature was identified from cross-sectional, retrospective public data sets without direct clinician involvement in feature validation, and that prospective clinical evaluation remains a necessary next step before deployment. However, the methodological framework introduced here, particularly the maximin-optimized benchmarking pipeline and the SHAP-based cross-disease feature attribution, is a potential framework for clinical researchers to apply to prospective cohorts, providing the informatics infrastructure needed for such validation.

### Limitations

External validation was adequate only for CRC; CD and LC were exploratory only (tier 3). The extreme AUC range in CD metabolomics (0.00–1.00, *n* = 40) likely reflects overfitting. Although each task compared cases and controls within the same study and sequencing platform, residual batch effects and unmeasured confounders (for example, medication and diet) may still influence observed associations. The non-zero SHAP threshold for signature definition is inclusive; alternative thresholds may yield different signatures. Zero imputation may bias results for rare taxa. The cross-sectional design precludes causal inference. Metabolomics data sets were generated using LCMS with variable feature coverage and incomplete KEGG annotation, which may have reduced power to detect shared metabolic pathways across diseases. All three training cohorts are derived from Chinese populations; the only adequately powered external validation cohort (CRC: PRJEB6070) is French. Known geographic gradients in gut microbiome composition may influence the prevalence and detectability of identified species.

Future work should prioritize prospective multi-ethnic validation, systematic confounder control, experimental characterization of CAG:41, and longitudinal studies establishing temporal relationships.

## MATERIALS AND METHODS

### Data sources and participants

Species-level gut microbiome abundance profiles (1,990 metagenomic species pan-genomes) were obtained as pre-processed matrices from the Human Gut Microbiome Atlas (18). All data sets comprised case-control comparisons of disease versus healthy participants. To minimize technical confounding (batch effects), each disease-specific task used cases and controls drawn from the same original study and country and generated using the same sequencing platform within that study. Due to incomplete and inconsistent clinical metadata across data sets, we did not adjust models for clinical covariates. No matching of cases and controls was performed; original cohort designs were retained. Training cohorts included PRJEB10878 (CRC; China), PRJEB15371 (CD; China), and PRJEB6337 (LC; China). External validation cohorts included PRJEB6070 (CRC; France), PRJEB2054 (CD; Spain), and PRJEB38481 (LC; United Kingdom). Serum metabolomics data were obtained from Metabolomics Workbench (ST000284 for CRC; ST000899 for CD) (43) and a previously published LC cohort (44). No external validation cohorts were available for metabolomics.

Eligibility criteria and participant demographics were as defined in the original publications. Cohort-level summaries of age, sex, and BMI are provided in Table S3 where metadata were available. All samples available in the downloaded feature matrices were included unless excluded a priori for analysis scope; exclusions and final sample counts are summarized in Fig. S1 and Table 1. Raw data preprocessing steps are available in the referenced sources. This study is a secondary analysis of publicly available, de-identified data sets. All original studies were approved by their respective institutional ethics committees prior to data deposition.

**TABLE 1:** Data set characteristics

Disease	Omics	Source	Disease samples	Healthy samples	Total samples	Features (*n*)
Colorectal cancer	Metagenomics	PRJEB10878	74	54	128	1,990
Liver cirrhosis	Metagenomics	PRJEB6337	123	114	237	1,990
Crohn’s disease	Metagenomics	PRJEB15371	67	55	122	1,990
Colorectal cancer	Metagenomics	PRJEB6070	53	61	114	1,990
Liver cirrhosis	Metagenomics	PRJEB38481	90	0	90	1,990
Crohn’s disease	Metagenomics	PRJEB2054	4	14	18	1,990
Colorectal cancer	Metabolomics	ST000284	64	84	148	113
Liver cirrhosis	Metabolomics	Published study	874	29	903	137
Crohn’s disease	Metabolomics	ST000899	20	20	40	586

### Sample size

Sample sizes were determined by availability of public data sets (Table 1). The CD metabolomics cohort (*n* = 40; 20 versus 20) and CD metagenomics external validation cohort (4 cases and 14 control samples) were notably small, and results from these data sets should be interpreted with particular caution.

### Configuration space

We exhaustively evaluated all combinations of preprocessing and modeling decisions across four categories. Preprocessing included four scaling strategies: no scaling, standard scaling [(*x* − μ)/σ], robust scaling [(*x* − median)/IQR], and logarithmic scaling (log1p-style transformation). Feature selection used five methods (variance thresholding, analysis of variance *F*-tests, mutual information estimation, random forest importance, and Mann-Whitney *U* test with Benjamini-Hochberg correction), each at three retention levels (10%, 30%, 50%), plus a no-selection baseline, yielding 16 feature selection options and 6 methods (considering no selection option as baseline method). Class imbalance was addressed using no balancing, SMOTE, or random undersampling. Classification used six algorithms: random forest, extreme gradient boosting (XGBoost), light gradient boosting machine (LightGBM), logistic regression, support vector machines with radial basis function kernel (SVM-RBF), and MLP. This resulted in a total of 1,152 configurations (4 preprocessing × 16 feature selection × 3 imbalance × 6 classification methods). Missing values were imputed with zero, consistent with the assumption that undetected species are absent or below detection limit; alternative imputation strategies were not evaluated and represent a limitation. The outcome for all tasks was binary disease status (disease versus healthy control) as defined in the original studies. Outcome ascertainment methods varied by cohort (clinical diagnosis, histopathology, or endoscopy as described in source publications).

### Nested cross-validation

Nested cross-validation ensured unbiased performance estimation and prevented data leakage. The outer loop employed fivefold stratified cross-validation for performance evaluation, while the inner loop employed threefold stratified cross-validation for hyperparameter optimization via exhaustive grid search. All preprocessing steps, including SMOTE and random undersampling where applied, were fitted exclusively within training folds and applied without refitting to held-out test folds to prevent data leakage. Hyperparameter grids are detailed in Supplemental Methods.

### Maximin robustness optimization

We selected configurations (*c*) maximizing minimum area under the receiver operating characteristic curve (AUC) across all six tasks (*t*).


$$c^{*}=\underset{c}{arg\;max}\left[\;\underset{t\in \{1,\ldots ,6\}}{min}\;{AUC}_{c,t}\right]$$

When multiple configurations achieved identical minimum AUC, we selected that with the highest maximum AUC. The optimal configuration comprised XGBoost, standard scaling, random forest feature selection, and no class balancing (minimum AUC = 0.882). Feature retention fraction (10%, 30%, or 50%) varied by disease to accommodate biological differences in dysbiosis architecture. This pipeline was used for all downstream analyses. Alternative selection criteria, including mean-AUC selection, median-AUC selection, and pareto-optimal multi-objective selection, were also evaluated across all configurations present in all six tasks. Per-task AUC profiles for all strategies are reported in Table S1.

### Statistical validation

AUC (primary), accuracy, precision, recall, specificity, and F1 score were computed on held-out folds (mean ± SD across fivefolds). Permutation testing (1,000 iterations) confirmed performance exceeded chance (45). For external validation, models trained on Chinese cohorts were applied to European cohorts without retraining. Bootstrap 95% confidence intervals (1,000 resamples, stratified) were computed for all external validation AUC estimates. For LC metabolomics (874 disease, 29 healthy), repeated downsampling to 1:1 and 3:1 case:control ratios (*N* = 100 iterations each) was performed using the maximin pipeline to assess performance stability under varying imbalance conditions.

### SHAP-based feature attribution and cross-disease signature

SHAP quantified feature contributions (46). The maximin pipeline was trained on complete training data sets using optimal hyperparameters from nested cross-validation. SHAP values were computed using TreeExplainer.

The cross-disease signature comprised species with non-zero SHAP in all three diseases (19 species). For validation, models were retrained using only signature species with the selected optimal configuration (feature selection = none) and retention computed as


$$Retention\;\%=\frac{AUC_{signature}}{AUC_{full}}\times 100$$

### Univariate discriminative analysis

To characterize disease-specific dysbiosis architecture, we evaluated the individual discriminative capacity of 1,990 microbial species across three diseases. For each species in each cohort, we performed fivefold stratified cross-validation: within each fold, species abundance was *z*-score normalized using training set parameters (StandardScaler), and a logistic regression classifier (L2 regularization, C = 1.0, sklearn default) was trained to predict disease versus healthy status. The area under the receiver operating characteristic curve (AUC) was computed on held-out samples, with mean AUC across folds quantifying univariate performance. Species with fewer than 10 valid abundance measurements or exhibiting zero variance (standard deviation < 10^−^³⁰) were excluded, yielding valid AUC estimates for CD (*n* = 1,042 taxa), LC (*n* = 1,251), and CRC (*n* = 1,252). To assess distributional architecture, we computed skewness and visualized the distribution on log scale to emphasize high-performing tail structure.

### Pathway analysis

Fisher’s exact test with Benjamini-Hochberg correction (alpha = 0.05) validated pathway enrichment using KEGG annotations from the Human Gut Microbiome Atlas (18) for metagenomics. For metabolomics, metabolites were mapped via KEGG REST API (47); nominal *P* values (*P* < 0.05) are reported where limited coverage precluded correction. Cross-omics convergence identified pathways enriched in both layers across all diseases. For metagenomics, SHAP-weighted pathway importance quantified each pathway’s contribution to disease prediction.

For pathway *p*


$$SHAP\;\%=\frac{\sum_{f\in \left(M_{p}\cap D\right)}{\text{SHAP}}_{f}}{\sum_{f\in D}{\text{SHAP}}_{f}}\times 100$$

where *D* is the set of selected disease-associated microbes, *M*_*p*_ is the set of background microbes annotated to pathway *p*, and SHAP_*f*_ is the aggregated SHAP value for microbe *f.*

Due to limited coverage of metabolomics, SHAP-weighted pathway importance couldn’t be quantified.

### Preprocessing effect analysis

To assess cross-task consistency of preprocessing effects, we computed SNR using a two-stage approach. First, within each task, AUC scores were *z*-transformed to account for varying difficulties; we then computed the mean *z*-score for each model × preprocessing pair, representing that method’s task-specific effect. Second, across all tasks, we calculated the mean (μ*z*) and standard deviation (σ*z*) of these per-task effects as


$$SNR=\frac{\mu z}{\sigma z}$$

This metric quantifies effect reliability: SNR > 1 indicates consistent improvement across tasks, SNR < −1 indicates consistent harm, while |SNR| < 1 suggests variable or task-dependent effects.

## Supplementary Material

Reviewer comments

## Data Availability

Input data sets are publicly available: microbiome data from the Human Gut Microbiome Atlas (PRJEB10878, PRJEB15371, PRJEB6337, PRJEB6070, PRJEB2054, PRJEB38481); metabolomics from Metabolomics Workbench (ST000284, ST000899), and the published LC cohort (44). Analysis code, pre-processed data, and results are available at https://github.com/sahukartika/robustness-first-XAI-Gut-Liver. STROME checklist is available at https://doi.org/10.5281/zenodo.19496855.

## AFTER EPUB

*[This article was published on 14 September 2026 with an error in Acknowledgments. The Acknowledgments were corrected in the current version, posted on 6 October 2026.]*

## SUPPLEMENTAL MATERIAL

Supplemental Figures msystems.00836-26-s0001.pdf Figures S1 to S5.

Supplemental Methods msystems.00836-26-s0002.docx Hyperparameters used in pipeline.

Table S1 msystems.00836-26-s0003.xlsx Comparison of optimization methods.

Table S2 msystems.00836-26-s0004.xlsx Covariate sensitivity analysis.

Table S3 msystems.00836-26-s0005.xlsx Sample metadata.

OPEN PEER REVIEW reviewer-comments.pdf An accounting of the reviewer comments and feedback.

ASM does not own the copyrights to Supplemental Material that may be linked to, or accessed through, an article. The authors have granted ASM a non-exclusive, world-wide license to publish the Supplemental Material files. Please contact the corresponding author directly for reuse.
