# Supplementary material for: A robustness-first cross-disease framework supports a candidate shared microbial redox axis and disease-specific metabolic divergence in colorectal cancer, Crohn’s disease, and liver cirrhosis
Source: mSystems. 2026 Sep 14;11(10):e00836-26. doi: 10.1128/msystems.00836-26 (PMC13712840; doi:10.1128/msystems.00836-26)
Supplement: Supplementary file 1 [file msystems.00836-26-s0001.pdf]

Supporting Information

A Robustness-First Cross-Disease Framework Identifies a Universal Microbial Redox Axis and Disease-Specific Metabolic Divergence in Colorectal Cancer, Crohn's Disease, and Liver Cirrhosis

Kartika Sahu[a,b], Palok Aich[a,b,*]

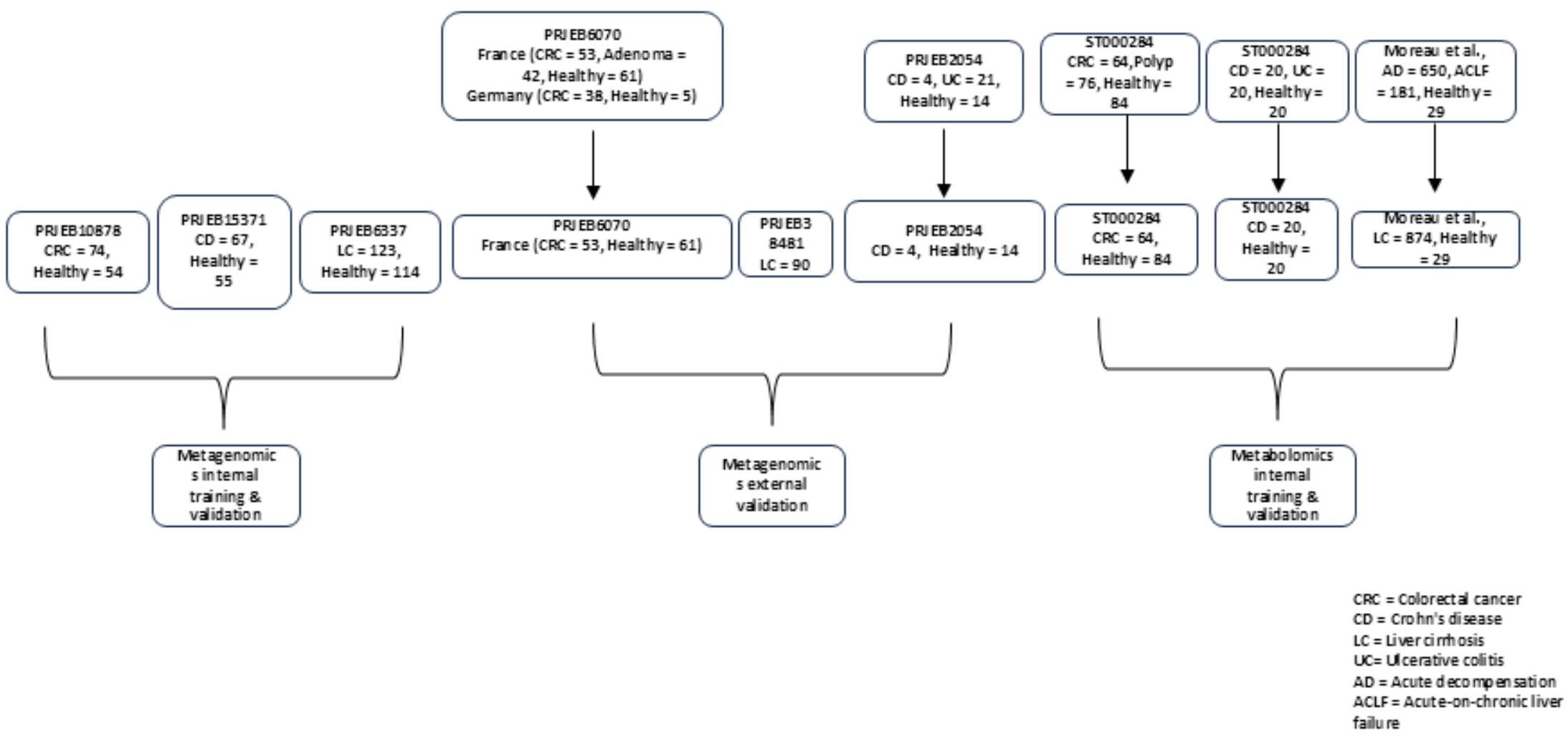

Figure S1. Sample flow and exclusions across cohorts and omics layers.
Flow diagram showing the number of samples available in each source dataset, the number retained for analysis, and exclusions applied a priori to match the scope of each binary classification task. Exclusions include removal of non-target disease groups (for example, ulcerative colitis samples from the Crohn's disease metabolomics dataset where present).

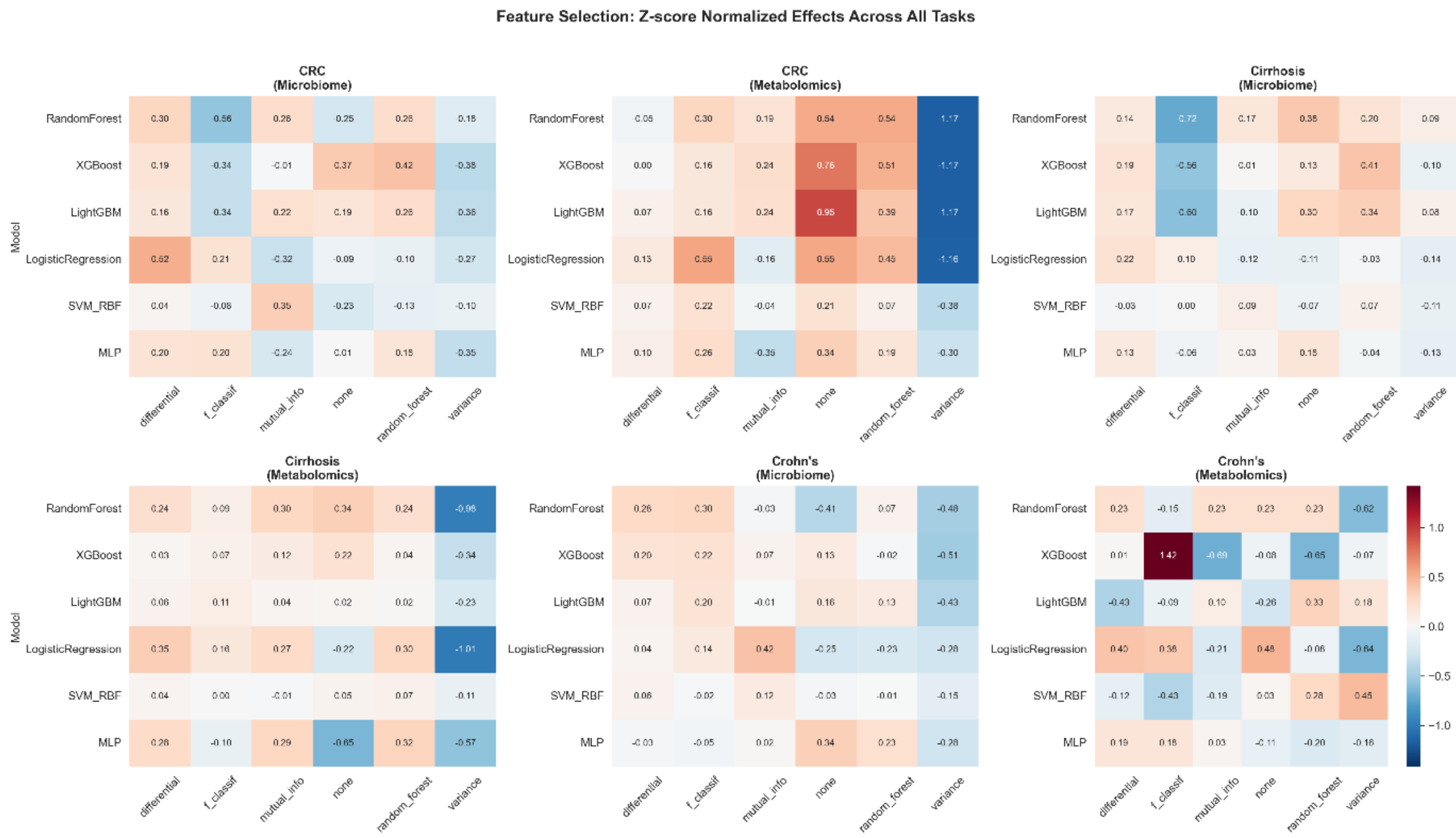

Figure S2: Heatmap showing signal-to-noise ratio (SNR) analysis of model-feature selection method interactions across tasks. Rows represent machine learning models; columns represent feature selection methods (No Selection, Variance Threshold, ANOVA F-test, Mutual Information, Random Forest Importance, Mann-Whitney U test) evaluated at three retention levels (10%, 30%, 50%). Color intensity indicates SNR magnitude (red = positive SNR, consistent improvement; blue = negative SNR, consistent degradation).

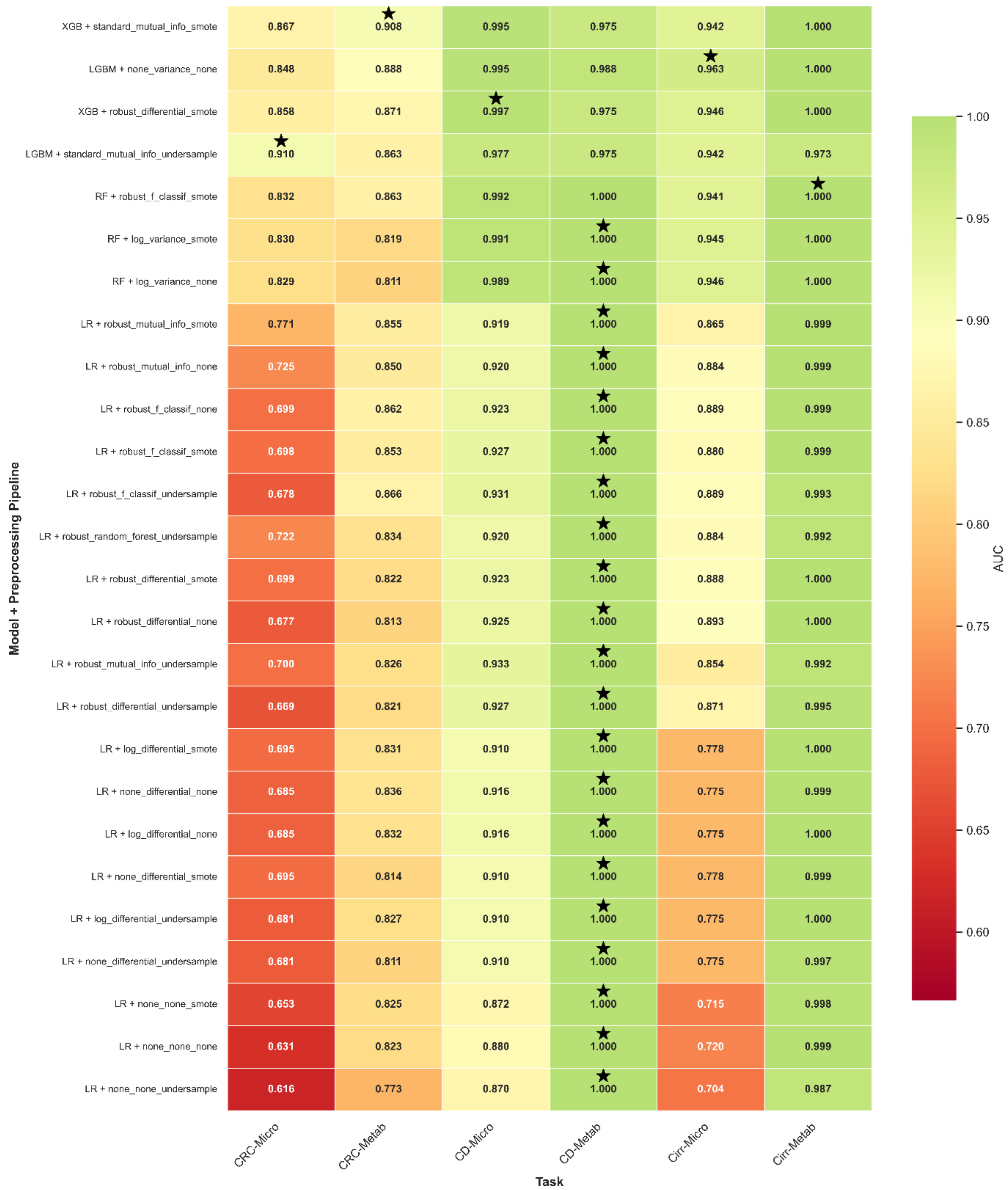

Figure S3: Heatmap of performance of best configs of each task across the task showing no config performed best in all tasks. Cell colour follows a red–yellow–green gradient (darker green = higher AUC). For each of the six classification tasks (columns), the (model, preprocessing-pipeline) pair achieving the highest mean AUC was selected; ties were broken by mean F1 score, and any remaining ties were retained. Each selected pair is marked with a black star (★) in the column of the task for which it was chosen.

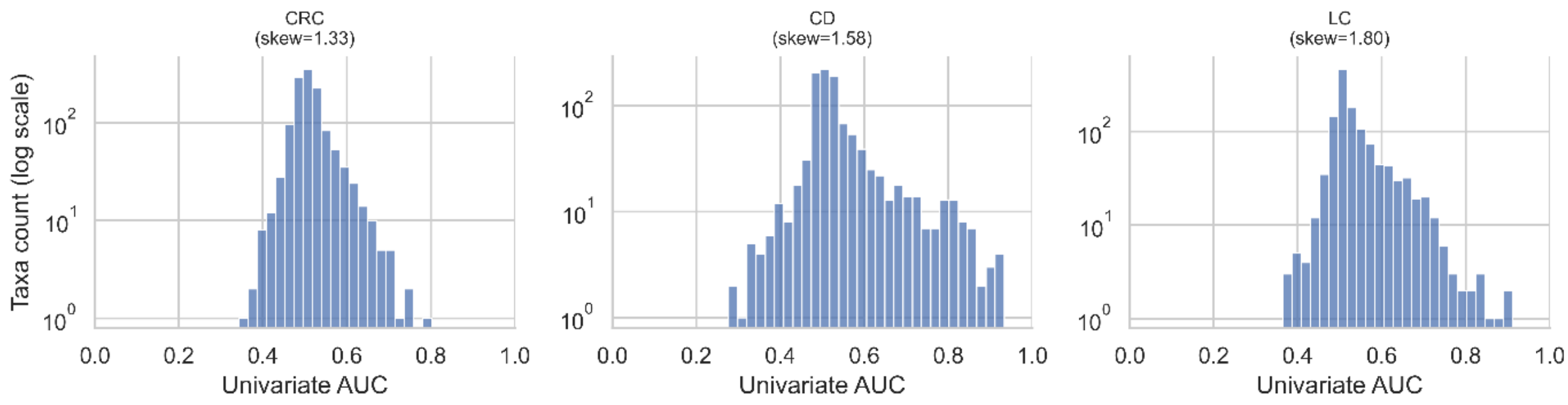

Figure S4: Dominant vs distributed signature of diseases with their skewness

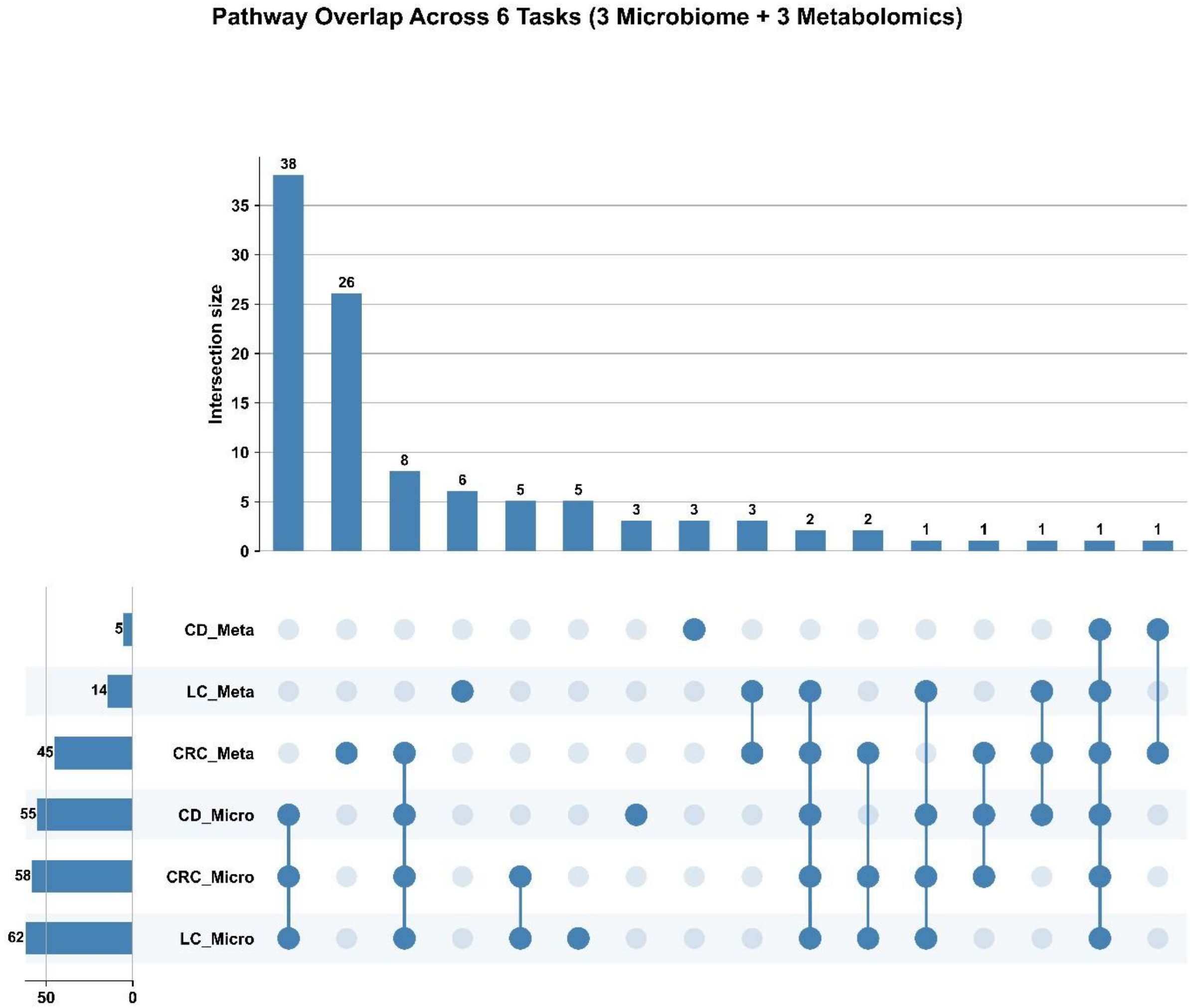

Figure S5: UpSet plot showing convergence of enriched both omics pathways across diseases. Horizontal bars (left) indicate total number of enriched pathways per disease. Vertical bars show pathway intersection sizes; dots and connecting lines indicate which diseases share each intersection. The intersection represents 1 pathway shared across all three diseases (CRC ∩ CD ∩ LC), which is alanine, aspartate, and glutamate metabolism.
