# Supplementary material for: A robustness-first cross-disease framework supports a candidate shared microbial redox axis and disease-specific metabolic divergence in colorectal cancer, Crohn’s disease, and liver cirrhosis
Source: mSystems. 2026 Sep 14;11(10):e00836-26. doi: 10.1128/msystems.00836-26 (PMC13712840; doi:10.1128/msystems.00836-26)
Supplement: Supplementary file 3 [file msystems.00836-26-s0002.docx]

Supporting Information

**A Robustness-First Cross-Disease Framework Identifies a Universal Microbial Redox Axis and Disease-Specific Metabolic Divergence in Colorectal Cancer, Crohn's Disease, and Liver Cirrhosis**

Kartika Sahu^a,b^, Palok Aich^a,b,*^

Supplementary Method S1

**Hyperparameter Optimization Grids**

Hyperparameters were optimized via exhaustive grid search using 3-fold cross-validation within each outer training fold:

**Random Forest:**

- n_estimators: [100, 300]
- max_depth: [10, 20, None]
- min_samples_split: [2, 10]
- max_features: ['sqrt', 'log2']
- class_weight: ['balanced', None]

**XGBoost:**

- n_estimators: [100, 300]
- max_depth: [3, 6]
- learning_rate: [0.01, 0.1]
- subsample: [0.8, 1.0]
- colsample_bytree: [0.8, 1.0]
- reg_alpha: [0, 0.1]
- reg_lambda: [1, 10]

**LightGBM (5 tasks):**

- n_estimators: [100, 300]
- max_depth: [3, 6]
- learning_rate: [0.01, 0.1]
- num_leaves: [31, 63]
- subsample: [0.8, 1.0]
- colsample_bytree: [0.8, 1.0]
- reg_alpha: [0, 0.1]
- reg_lambda: [1, 10]

**LightGBM (CD metabolomics only):** Due to small sample size (n=40), the default min_child_samples=20 parameter exceeded training fold sizes. The grid was expanded:

- min_child_samples: [2, 5, 10]
- min_data_in_leaf: [1, 3, 5]
- All other parameters unchanged

**Logistic Regression:**

- C: [0.1, 1.0, 10.0]
- penalty: ['l1', 'l2']
- solver: 'liblinear'
- class_weight: ['balanced', None]

**SVM-RBF:**

- C: [0.1, 1.0, 10.0]
- gamma: [0.001, 0.01, 0.1]
- class_weight: ['balanced', None]

**Multi-Layer Perceptron:**

- hidden_layer_sizes: [(50,), (100,), (100, 50)]
- activation: ['relu', 'tanh']
- alpha: [0.0001, 0.001, 0.01]
- learning_rate_init: [0.001, 0.01]
- max_iter: 1000

Best hyperparameters from the first outer fold were saved and used for SHAP analysis.
